# CRISPR-based antimicrobials to obstruct antibiotic-resistant and pathogenic bacteria

**DOI:** 10.1371/journal.ppat.1009672

**Published:** 2021-07-08

**Authors:** Dennise Palacios Araya, Kelli L. Palmer, Breck A. Duerkop

**Affiliations:** 1 Department of Biological Sciences, University of Texas at Dallas, Richardson, Texas, United States of America; 2 Department of Immunology and Microbiology, University of Colorado School of Medicine, Aurora, Colorado, United States of America; Nanyang Technological University, SINGAPORE

## Introduction

The rapid emergence of antibiotic-resistant bacteria and the comparatively limited development of new antibacterial molecules pose a major threat to modern medicine by jeopardizing our ability to treat and prevent infections [1]. The overuse of antibiotics in both medical and agricultural settings has facilitated the emergence of multidrug-resistant (MDR) bacteria [2]. Furthermore, many antibiotics lack specificity, indiscriminately killing pathogenic and nonpathogenic bacteria and contributing to antibiotic-associated infections [3,4]. This highlights the critical need for novel therapeutics that circumvent existing modes of drug resistance while adding specificity [5]. Toward this goal, clustered regularly interspaced short palindromic repeats/CRISPR-associated protein (CRISPR/Cas) systems are being explored as possible antimicrobials to target bacteria and/or antibiotic resistance genes in a sequence-specific manner. These promising targeted therapies are the focus of synthetic biology companies such as Locus Biosciences, Intellia Therapeutics, and Eligo Bioscience, to name a few. Although these technologies are mostly in the preclinical phase, the main goal is to provide precision antimicrobials for infectious diseases. Here, we discuss the present state of CRISPR antimicrobial research, with a specific focus on the biological differences between targeting plasmids versus chromosomes, the efficacy of CRISPR antimicrobials in infection and colonization models, and the various delivery mechanisms for these potential therapeutic tools.

### Sequence-specific targeting by CRISPR/Cas

**Fig 1:**
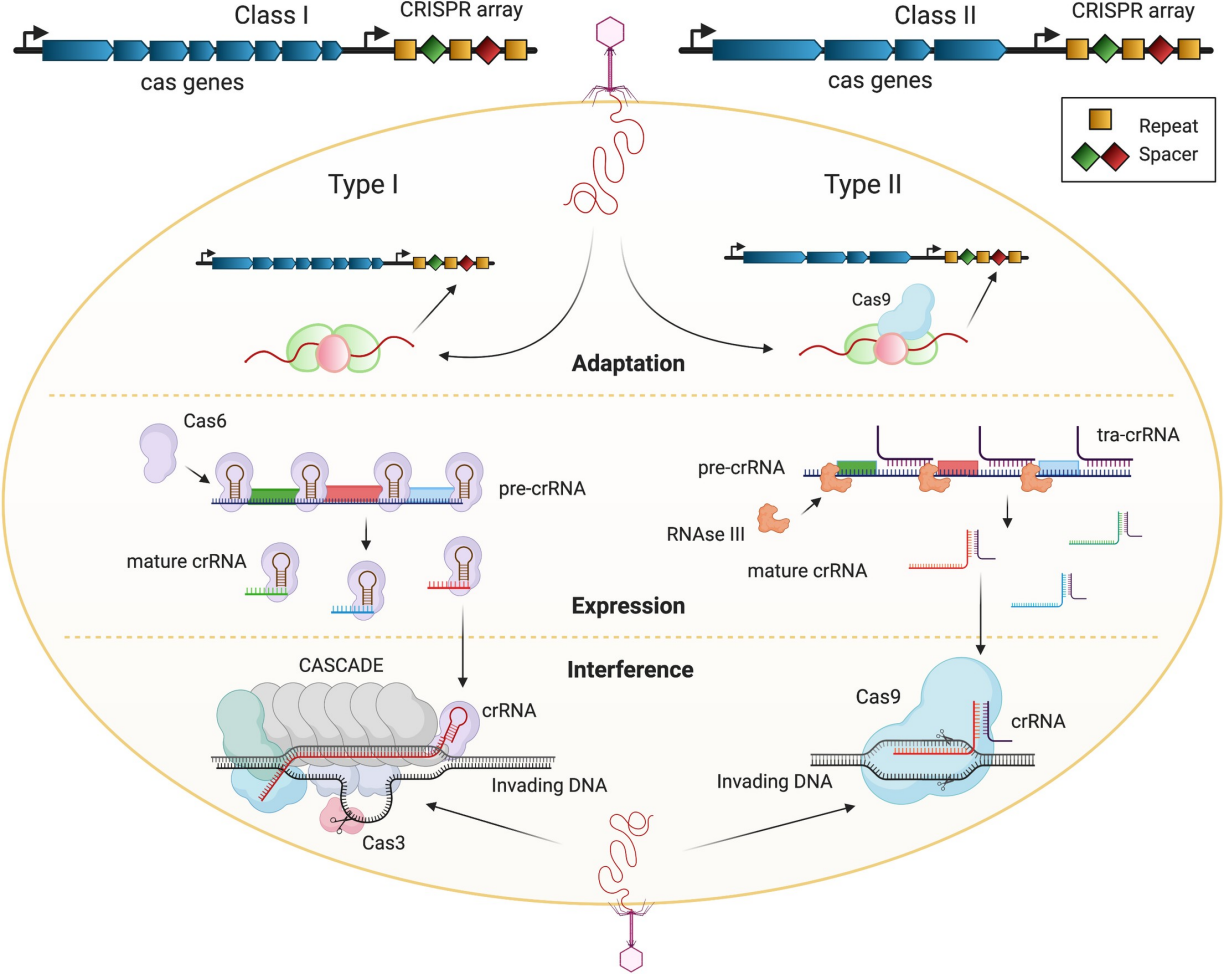
Mechanisms of action of CRISPR/Cas immunity. Left: Class I CRISPR/Cas systems (modeled by Type I system) utilize a multi-subunit complex, termed “Cascade,” as the effector machinery. Right: Class II CRISPR/Cas systems (modeled by Type II system) use a single effector protein (such as Cas9) for interference. Both classes also consist of spacer (diamonds) and repeat (squares) arrays. Top: During adaptation, the Cas1–Cas2 complex takes a sequence of the invading DNA and integrates it into the CRISPR array as a novel spacer. Center: In the next stage, termed “expression,” the CRISPR array is transcribed into pre-crRNAs that are further processed into mature interfering crRNAs. Bottom: During the interference stage, the mature crRNAs guide the Cas proteins to their DNA target. Upon binding of the crRNA to their cognate DNA target, the Cas protein generates a double-stranded DNA break in the target. Created with BioRender.com. Cascade, CRISPR-associated complex for antiviral defense; CRISPR/Cas, clustered regularly interspaced short palindromic repeats/CRISPR-associated protein.

CRISPR/Cas systems are adaptive defense mechanisms against mobile genetic elements (MGEs). A key hallmark of these systems is the CRISPR array, a genomic locus comprised of unique spacers alternated by identical repeats [6]. Effector proteins function as interference molecules to silence foreign genetic elements [6]. During adaptation, some CRISPR types integrate a short fragment of foreign DNA into the CRISPR array, thereby providing a genetic memory of MGE invasion, referred to as a spacer (Fig 1). Transcription of the CRISPR array yields precursor CRISPR RNAs (pre-crRNAs) that are enzymatically processed into mature crRNAs (Fig 1). Upon invasion of bacteria by MGEs with complementary sequence, crRNAs guide effector proteins to these targets for enzymatic cleavage, ultimately causing sequence-specific elimination of the invading molecule [6] (Fig 1).

CRISPR/Cas systems have been grouped into 2 classes and further classified into 6 subtypes (I to VI). Types I, III, and IV utilize multi-Cas protein complexes for interference, while Types II, V, and VI employ a single effector protein (Fig 1) [6]. Type I CRISPR/Cas systems utilize a guide RNA-bound multi-subunit complex termed “CRISPR-associated complex for antiviral defense (Cascade),” combined with an effector nuclease, Cas3. Establishment of the guide RNA:DNA target complex stabilizes Cascade, allowing the recruitment of Cas3 and the subsequent exonucleolytic cleavage of the DNA target (Fig 1) [6]. Type II CRISPR/Cas systems encode the dual RNA-guided endonuclease, Cas9. Once loaded with a mature crRNA, Cas9 recognizes and binds a complementary target sequence by DNA:RNA base pairing. If the complementary base pairing between the crRNA and the target is sufficient, Cas9 generates a double-stranded DNA break in the target [6] (Fig 1). While Type I and Type II CRISPR systems are the most widely used as CRISPR antimicrobials, as they are amenable to genetic modifications and their mechanism of action are well studied, the Type VI system was recently proposed as an alternative because it inhibits bacterial growth when targeting a plasmid or the chromosome [7].

### CRISPR antimicrobials: An overview

**Fig 2:**
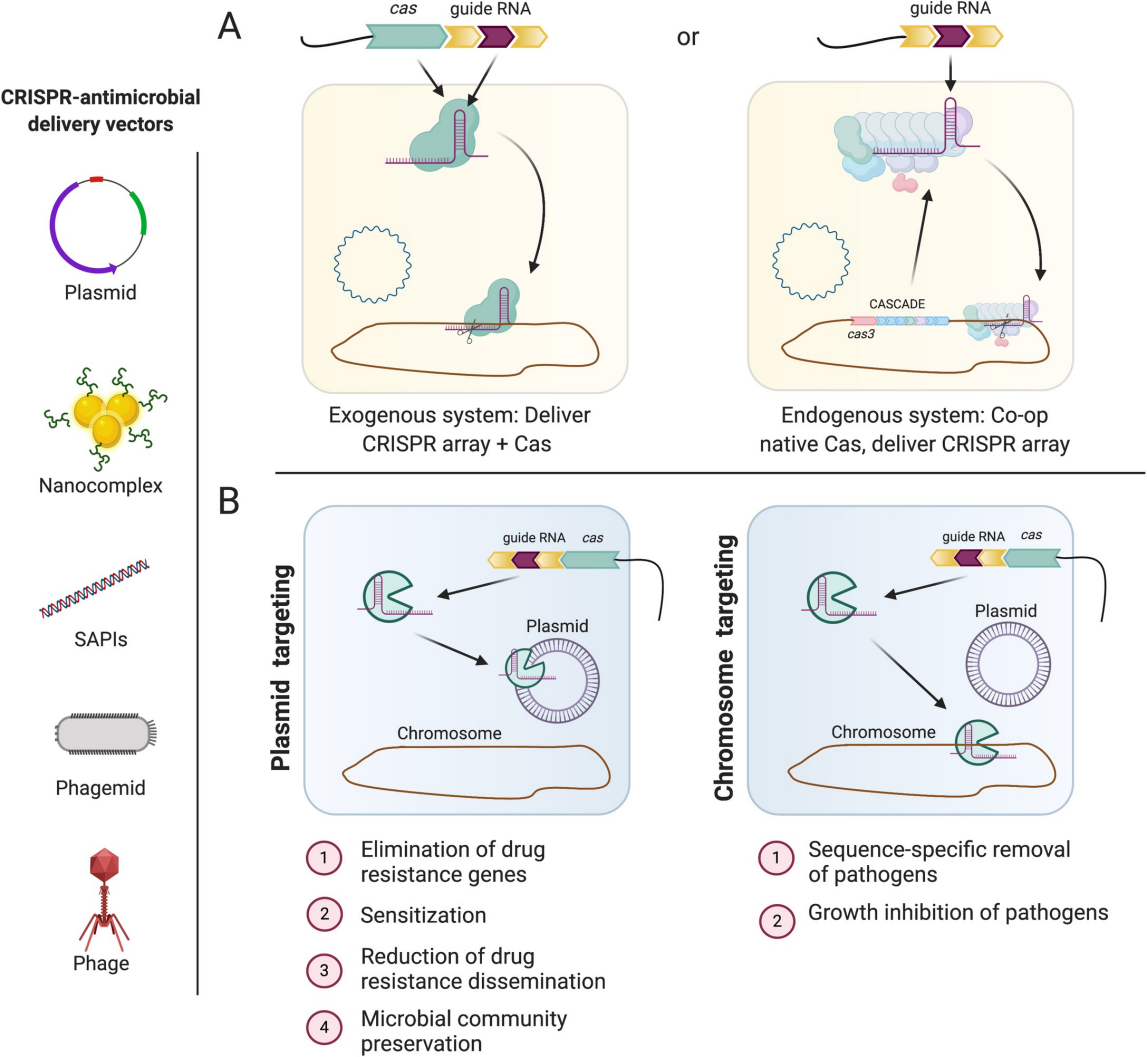
Strategies for the development of CRISPR-based antimicrobials. **(A)** One approach is to deliver a complete exogenous CRISPR/Cas system to target a desired locus (chromosome or plasmid). Alternatively, if the target pathogen harbors a CRISPR/Cas system, one can deliver a CRISPR array and exploit the native Cas proteins to drive DNA targeting. **(B)** CRISPR antimicrobials can be designed to target a chromosomal locus, leading to cell death or a plasmid locus, leading to plasmid loss and sensitization of the target bacteria. Left: an array of different CRISPR antimicrobial delivery vehicles proposed by different research groups. Created with BioRender.com. Cascade, CRISPR-associated complex for antiviral defense; CRISPR/Cas, clustered regularly interspaced short palindromic repeats/CRISPR-associated protein; SAPI, staphylococcal pathogenicity island.

CRISPR/Cas systems can be engineered to target nearly any sequence of interest, which has led to a genome editing revolution. CRISPR/Cas systems have also been repurposed as potential antimicrobials, where removal of undesirable genetic traits in microorganisms has been the focus [8]. Two different approaches have been proposed for CRISPR-based antimicrobials: co-opting endogenous “native” systems in bacteria to deliver self-targeting CRISPR arrays or delivering a complete exogenous “foreign” self-targeting system to bacteria (Fig 2A) [9].

A key characteristic of CRISPR/Cas is its target specificity, which allows for discrimination between commensal and pathogenic bacteria. Guide RNAs can be engineered to target antibiotic resistance, virulence, or essential genes specific to pathogens [10]. Depending on experimental design, targeting outcomes include cell death or growth inhibition of the targeted bacterium, targeted deletion of genes from pathogens, loss of mobile elements such as antibiotic resistance plasmids, or transcriptional repression of targeted gene(s) (Fig 2B). The location of the target sequence (chromosome or plasmid) and whether the target encodes an essential function are critical factors [11] (Fig 2B). In contrast to eukaryotic genomes that tolerate CRISPR/Cas9 cleavage by initiating repair mechanisms [12], cleavage of bacterial chromosomes at any location is usually lethal [11,13–20]. Additionally, other studies have demonstrated the sensitization of drug-resistant pathogens by directly targeting plasmids harboring antibiotic resistance genes. This strategy leads to plasmid curing, thereby reducing the prevalence of antibiotic-resistant bacteria [11,21–27]. Researchers have also utilized a catalytically “dead” Cas9 (dCas9) to suppress transcription of the methicillin resistance gene *mecA* in *Staphylococcus aureus* [28] and the class 1 integron in *Escherichia coli*, which is associated with a variety of resistance genes [29]. The diversity of these applications demonstrates how valuable CRISPR/Cas systems could become in the fight against rapidly emerging MDR bacteria.

### CRISPR antimicrobials to confer bacterial cell death: Select studies

The groundbreaking work of Edgar and Qimron led to the first discoveries of how self-targeting capabilities could be engineered into a CRISPR/Cas system. They modified the *E*. *coli* Type I-E CRISPR system to target an endogenous prophage, showing that 98% of self-targeted bacteria were killed [30]. More recently, the native Type I-B CRISPR/Cas3 system of *Clostridioides difficile* was repurposed for self-targeting using a recombinant bacteriophage to deliver a chromosome-targeting CRISPR. By comparing the killing efficacy of *C*. *difficile* bacteriophages with or without a CRISPR payload, they demonstrated that addition of a self-targeting CRISPR improved bacterial killing in vitro. Using a mouse model of *C*. *difficile* intestinal infection, vegetative *C*. *difficile* numbers were reduced by approximately 10-fold when mice were gavaged with CRISPR-enhanced bacteriophages [16]. Modified exogenous CRISPR/Cas systems have also been deployed for sequence-specific killing of bacteria. For example, engineered Type II CRISPR/Cas9 of *Streptococcus pyogenes* was successfully deployed to target chromosomally encoded antibiotic resistance genes in *E*. *coli* and *S*. *aureus*, causing cell death in in vitro cultures as well as in in vivo models [11,21] (Fig 2A).

While these studies achieved reduced bacterial burden using engineered CRISPR/Cas genome-targeting constructs, they also reported the emergence of escape mutants that avoided cell death. For instance, some studies described recombination and deletions that resulted in inactivation of the CRISPR loci and elimination of *cas* genes and target sequences, to name a few [11,13,16,21]. This raises the important question of whether CRISPR antimicrobials would encounter a similar fate when used in vivo to treat bacterial infections. Contrary to antibiotics, which can cause dysbiosis and fuel the spread of resistant bacteria, CRISPR antimicrobials kill only a small proportion of the bacterial population, likely allowing other community members to occupy the niche and restrict the growth of CRISPR-resistant bacteria [21]. Although, to date, no in vivo studies have fully explored this idea. Additionally, some countermeasures have been suggested to combat CRISPR antimicrobial “escape” by pathogens. For instance, reduction of the CRISPR array to a single spacer repeat could prevent recombination between repeats and subsequent spacer deletion [13], and overexpression of *cas9* has been shown in *Enterococcus faecalis* to increase lethality of CRISPR self-targeting [31].

### CRISPR antimicrobials to cure resistance plasmids

Plasmids are attractive targets for the elimination of drug resistance genes (Fig 2B). Pioneering studies have utilized engineered CRISPR/Cas9 to target antibiotic resistance plasmids, demonstrating that their targeting does not result in bacterial cell death but rather in antibiotic sensitization via plasmid loss [11,21]. To improve overall efficacy, approaches such as applying positive selection for antibiotic-sensitized cells may be implemented. For example, a recent study utilized a bacteriocin-encoding plasmid, pPD1, to deliver a CRISPR/Cas9 programmed to target antibiotic resistance genes in *E*. *faecalis* [25]. Uptake of the engineered plasmid by antibiotic-resistant recipient bacteria led to loss of targeted resistance plasmids while simultaneously providing immunity against the bacteriocin. Recipient cells that did not acquire the engineered plasmid were killed by the bacteriocin, thereby indirectly selecting for cells that lack antibiotic resistance [25].

Antibiotic resistance genes are commonly encoded on self-transmissible high-copy plasmids [11]. To determine whether CRISPR/Cas can eradicate all plasmid copies, Tagliaferri and colleagues used the CRISPR/Cas9 system to target a β-lactamase gene in *E*. *coli* strains harboring either high or low copy number plasmids. Complete eradication of the low copy number plasmid but not of the high copy number plasmid was observed [32]. Importantly, this study highlights challenges posed by targeting high-copy plasmids and the need to optimize the system to completely clear antibiotic resistance genes encoded by these types of plasmids.

Although there are many advantages of exploiting CRISPR/Cas systems for plasmid curing, the strategy has limitations. Targeting a plasmid can select for undesirable recombination events in the targeted region. In many cases, antibiotic resistance genes are flanked by transposases or recombinases; thus, directly targeting these genes for the purpose of curing plasmids is not recommended because of the added risk of selecting for new plasmid variants or for transposition of the resistance cassette into the chromosome [23]. Additionally, plasmid addiction systems such as toxin/antitoxins encoded by the targeted plasmid could inadvertently cause cell death secondary to plasmid curing [11].

### Delivery of CRISPR antimicrobials to targeted bacterial cells: Approaches and considerations

A major challenge in the deployment of CRISPR antimicrobials is the need to develop robust delivery systems. Two early studies utilized phage capsids as delivery vehicles. One group constructed a phagemid based on the temperate phage phiNM1 [21], while the other designed a phagemid system based on the M13 phage [11]. Each group used this approach to successfully deliver Cas9 and guide RNAs targeting virulence genes into *S*. *aureus* [21] or *E*. *coli* [11]. Another team expanded on these studies by designing a temperate delivery phage with increased host range, which was accomplished by diversifying the phage tail fiber protein [17]. Alternatively, other groups have proposed nonviral delivery approaches. Mobile staphylococcal pathogenicity islands (SAPIs) have been adapted as potential vectors. Development of these vectors consisted of replacing the SAPIs’ toxin genes with CRISPR/Cas9 cargos [14]. The effectiveness of the SAPI antibacterial was also demonstrated in a murine subcutaneous infection model [14]. Low packaging efficiencies of CRISPR/Cas cargos and the evolution of phage resistance in targeted bacteria present challenges for a broader use of these technologies. To circumvent low packaging efficiencies, Kang and colleagues effectively delivered a nanocomplex of polymer-derivatized Cas9 and a guide RNA (Cr-nanocomplex) designed to target the methicillin resistance gene *mecA* in *S*. *aureus* [18]. Finally, owing to their flexible host ranges, size and coding capacity, and cellular receptor independence, conjugative plasmids present an attractive delivery option [15,25,27]. However, low conjugation efficiencies and restriction systems that block plasmid transfer remain as considerable limitations.

### Efficacy of CRISPR antimicrobials in infection and colonization models

The development of CRISPR systems as selective and titratable antimicrobials requires further study to determine therapeutic efficacy. To date, only 9 studies have included in vivo infection or colonization models in their experimental design to test the efficacy of CRISPR-based antimicrobials [7,11,14,16,17,21,25,32,33]. Of those studies, only 2 have compared the efficacy of CRISPR/Cas antimicrobials to traditional antibiotics [11,21]. Using a mouse skin colonization model, one study showed that CRISPR/Cas9 targeting of *S*. *aureus* decreased *S*. *aureus* skin colonization significantly compared to other treatment conditions. In comparison, treatment with systemic streptomycin decolonized the mice completely of staphylococci [21]. Another study showed that carbenicillin treatment of *Galleria mellonella* infected with enterohemorrhagic *E*. *coli* was superior compared to treatment with a CRISPR antimicrobial [11]. Although more data are needed, these studies suggest that instead of using CRISPR antimicrobials as replacements for conventional antibiotics, they may be more effectively deployed in combination therapies. For example, in a 2-step experiment, *E*. *coli*–infected *G*. *mellonella* larvae were first treated with CRISPR/Cas9 targeting *bla*_*TEM-1*_, conferring beta-lactam resistance, then treated with ceftriaxone, resulting in the survival of 70% of dual-treated larvae compared to 30% of larvae treated with antibiotics alone [32]. In another example, CRISPR antimicrobials could be used for decolonization of antibiotic-resistant bacteria, rather than infection treatment. CRISPR targeting of antibiotic resistance genes between antibiotic treatments could potentially reduce the expansion of resistant bacteria following antibiotic usage. Only 1 study has tackled this question by using a mouse model of *E*. *faecalis* intestinal colonization. CRISPR/Cas9 targeting of the *E*. *faecalis* erythromycin resistance gene *ermB* significantly reduced the overall presence of erythromycin-resistant *E*. *faecalis* in the gut after antibiotic treatment [25].

## Conclusions

The public health crisis of antimicrobial resistance calls for novel approaches to fight bacterial infections. Over the past 10 years, CRISPR/Cas systems have been gaining attention as programmable sequence-specific antimicrobials capable of targeting nearly any sequence of interest. The unique specificity of CRISPR-based antimicrobials means that it would be possible to remove only specific bacteria or virulence traits from the population while otherwise leaving the microbiota intact, features that antibiotics lack. Using an array of different delivery methods such as phages and plasmids, CRISPR antimicrobials have been shown to be lethal when directed to the chromosome. Additionally, they can be used to sensitize bacteria to an antibiotic by eliminating plasmids harboring antibiotic resistance genes. Other potential uses of CRISPR/Cas such as targeted deletion of genes from pathogens and suppression of antibiotic resistance gene expression need to be further examined.

While this is a promising strategy, more studies utilizing in vivo models are necessary to establish the therapeutic efficacy of these antimicrobials compared to traditional antibiotics and to determine whether infections can be treated with CRISPR antimicrobials alone or if they need to be combined with antibiotics to improve their efficacy. In addition, determining what role they could play in reducing both antibiotic resistance dissemination and expansion of resistant bacteria in the gut following antibiotic treatment will be critical to fully understand the potential of these technologies.
