# Correction: CRISPR-based antimicrobials to obstruct antibiotic-resistant and pathogenic bacteria

**DOI:** 10.1371/journal.ppat.1010153

**Published:** 2021-12-13

**Authors:** Dennise Palacios Araya, Kelli L. Palmer, Breck A. Duerkop

There is an error in the sixth sentence of the first paragraph of the Introduction which should not include reference to Intellia Therapeutics. The correct sentence is: These promising targeted therapies are the focus of synthetic biology companies such as Locus Biosciences and Eligo Bioscience, to name a few.
